# Effects of combined abiotic stresses on nutrient content of European wheat and implications for nutritional security under climate change

**DOI:** 10.1038/s41598-022-09538-6

**Published:** 2022-04-05

**Authors:** Yamdeu Joseph Hubert Galani, Emilie Marie Øst Hansen, Ioannis Droutsas, Melvin Holmes, Andrew Juan Challinor, Teis Nørgaard Mikkelsen, Caroline Orfila

**Affiliations:** 1 School of Food Science and Nutrition, University of Leeds, Leeds, LS2 9JT UK; 2 Section of Natural and Applied Sciences, School of Psychology and Life Sciences, Canterbury Christ Church University, Canterbury, CT1 1QU UK; 3 Department of People and Technology, Roskilde University, 4000 Roskilde, Denmark; 4 School of Earth and Environment, University of Leeds, Leeds, LS2 9JT UK; 5 Department of Environmental and Resource Engineering, Technical University of Denmark, Bygningstorvet 115, 2800 Kgs, Lyngby, Denmark

**Keywords:** Plant sciences, Environmental sciences

## Abstract

Climate change is causing problems for agriculture, but the effect of combined abiotic stresses on crop nutritional quality is not clear. Here we studied the effect of 10 combinations of climatic conditions (temperature, CO_2_, O_3_ and drought) under controlled growth chamber conditions on the grain yield, protein, and mineral content of 3 wheat varieties. Results show that wheat plants under O_3_ exposure alone concentrated + 15 to + 31% more grain N, Fe, Mg, Mn P and Zn, reduced K by − 5%, and C did not change. Ozone in the presence of elevated CO_2_ and higher temperature enhanced the content of Fe, Mn, P and Zn by 2–18%. Water-limited chronic O_3_ exposure resulted in + 9 to + 46% higher concentrations of all the minerals, except K. The effect of climate abiotic factors could increase the ability of wheat to meet adult daily dietary requirements by + 6% to + 12% for protein, Zn and Fe, but decrease those of Mg, Mn and P by − 3% to − 6%, and K by − 62%. The role of wheat in future nutrition security is discussed.

## Introduction

Without action, climate change will impact nutrition through decreased food access and reduced dietary diversity^1,2^. Modelling studies have suggested that higher atmospheric CO_2_ would impact nutritional quality of staples globally, notably impacting protein^3^, Zn^4^, Fe^5^, and nutrients in general^6^. Wheat (*Triticum aestivum* and *T. durum*) is a widely cultivated crop, crucial to the food and nutrition security of populations worldwide. Global average daily supply of wheat and its products is estimated at 179 g per capita, providing 527 kcal, 15.8 of g protein and 2.4 g of fats^7^. It is consumed in all the continents, Europeans having the largest supply with 298.6 g/day, followed by Oceanians, Asians, and Americans. Africans have the lowest supply, estimated at 130.6 g/day^8^. Consuming 160 g of commercially prepared whole-wheat bread contributes 36% of adult daily protein requirements^9^. Similarly, consuming 200 g/day could meet 76% of Fe, 72–84% of Mg, 78% of Zn, 90% of Mn, > 100% of P, and 41% of K adult needs^10^. Wheat-based foods provide around 40% of dietary fiber intake and make significant contributions to the intake of B vitamins, Ca, Cu, Fe, Mg, Se and Zn in UK consumers^7^. Any change in the yield or nutrient content of wheat could have a large impact on nutrient intake and dietary health of millions of people.

Human activities such as industrialization and deforestation have led to sustained increases in atmospheric temperature, CO_2_ and O_3_ levels, as well as decreased availability of water for agriculture. These atmospheric conditions cause physiological stress responses affecting crop performance, grain yield and nutrient accumulation^11,12^. For instance, high temperature treatments (40/20 vs 25/20 °C day/night) ten days after anthesis reduced grain number, weight, and the content of polysaccharides and proteins in many wheat varieties^13^. Elevated concentration of atmospheric CO_2_ (546–586 ppm) lead to lower concentrations of protein, Zn, Fe in C_3_ grains such as wheat^14^. Högy et al.^15^ observed an increase of 1000-grain weight, but a decrease in proteins, amino acids, Fe and Ca when CO_2_ concentration was 150 ppm above the ambient value. Similarly, concentrations of grain protein, Fe, Zn, S and Ca were significantly reduced at elevated CO_2_ (550 ppm) as compared to the ambient 384 ppm^16^. Chronic elevated O_3_ (25–35 ppb) resulted in lower rubisco enzyme activity, chlorophyll, and photosynthetic rate in *T. aestivum* wheat, and significantly reduced sugar, starch, and protein contents in two wheat varieties^17^. A review of 42 experiments performed in Asia, Europe and North America showed that high O_3_ has a strong negative effect on 1000-grain weight, and weaker but significant negative effects on starch concentration and volume weight. Conversely, a significant increase of protein and several nutritionally important minerals (K, Mg, Ca, P, Zn, Mn, Cu) was observed, but yields were significantly decreased. Effects on Fe, S and Na were not significant, or results were inconclusive^18^. Climate change has led to significant geographical and seasonal redistribution in precipitation and rise in temperature^19^. In wheat, water deficit at grain filling stage reduces grain-filling duration and ultimately reduces grain number and size^20^. Water deficit also reduced grain yield, macro and micronutrient contents in common bean, triticale, and wheat^21^. Severe drought conditions caused a significant reduction in total protein and carbohydrates and a gradual augmentation in total fibers in wheat grains^22^. Contrarily, moderate drought during grain filling in wheat was found to increase grain protein content, although a slight decrease in grain yield was also observed^23^.

Simultaneous combinations of abiotic stresses may not result in additive effects on plant growth and productivity^24–26^. Wheat grown under higher temperature and CO_2_ conditions (700 ppm CO_2_ and 3 °C temperature rise) had significantly lower straw and grain yield, particularly due to severe reduction in number of spikes per plant, although supplied with ample fertilization^11^. Multifactor combination of ambient or elevated CO_2_ (385 and 700 ppm), O_3_ (20 and 60 ppb) and temperature (19/12 and 24/17 °C) showed a decrease in growth and production in oilseed rape and barley^24^. In contrast, a large decrease in wheat grain yield (− 43.6%) was observed under the additive effect of 50% water deficit stress and elevated O_3_ (+ 20 ppb), while individually applied water deficit stress and elevated O_3_ alone reduced grain yield by − 19.8% and − 17.9% respectively^27^. Response to abiotic stresses differs not only among crop species^14,24,28^, but also among crop varieties of the same species. Under elevated O_3_, more reduction of grain yield was observed in *T. aestivum* (− 15% and − 19%) as compared to *T. durum* (− 9% and − 13%)^17^. Similarly, the landrace variety of wheat was more sensitive to O_3_ than the modern varieties^29^. Very little consideration has been given to interaction of O_3_ with other abiotic factors on yield and nutrient content of plants. Besides, to our knowledge, no attempts have been made to measure to what extent combined climate change factors will affect the ability of crops, including wheat, to fulfil the nutritional requirements of the population.

We have used a multifactorial, controlled growth chamber experiment with combinations of CO_2_ (400 and 700 ppm), temperature (19/12 and 24/17 °C) and different O_3_ exposure regimes (5.9 –7.2 ppb and episodic or chronic 80 to 100 ppb) on three European wheat spring varieties. For context on how these conditions related to climate change, global CMIP6 projections indicate that by 2081–2100, scenario SSP1-2.6 has a CO_2_ concentration of approximately 450 ppm and a temperature increase (relative to 1995–2014) of approximately 1.8 °C; SSP5-8.5 has a CO2 concentration of approximately 1100 ppm and a temperature increase of approximately 4.4 degrees Celsius, rising to approximately a 5 °C increase by 2100^30^. We have systematically evaluated the effect of these combinations of abiotic stress conditions on yield, protein and dietary mineral nutrient content, and the interaction between these. We estimated the potential consequences on the nutrient supply from wheat. We discuss potential implications of climate change on nutrition security.

## Results

### Effect of combined abiotic factors on mineral content of wheat grain

**Figure 1:**
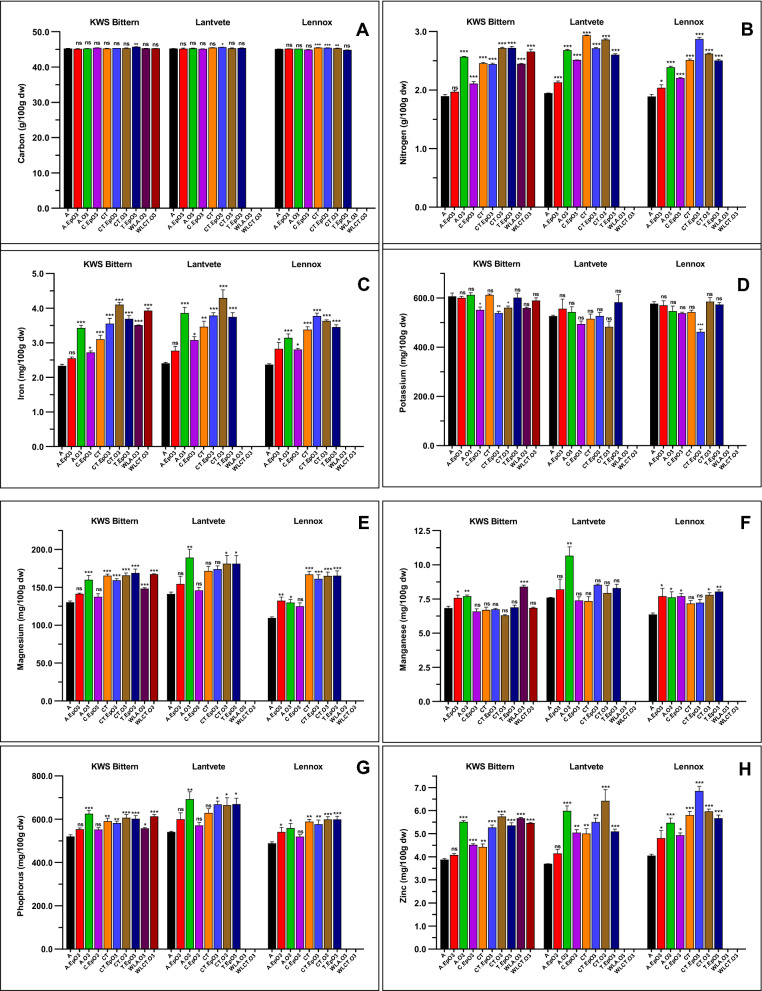
Effect of temperature, CO_2_, ozone and water availability on grain mineral content (carbon (**A**), nitrogen (**B**), iron (**C**), potassium (**D**), magnesium (**E**), manganese (**F**), phosphorus (**G**) and zinc (**H**)) of three European wheat varieties. Treatments: A = Ambient CO2, lower temperature settings and no O3 addition (control). A.EpO3 = Ambient CO2, lower temperature settings and episodic O3 addition. A.O3 = Ambient CO2, lower temperature settings and chronic O3 addition. C.EpO3 = High CO2, lower temperature settings and episodic O3 addition. CT = High CO2, higher temperature settings, and no O3 addition. CT.EpO3 = High CO2, higher temperature settings and episodic O3 addition. CT.O3 = High CO2, higher temperature settings and chronic O3 addition. T.EpO3 = Ambient CO2, higher temperature and episodic O3 addition. WLA.O3: Ambient CO2, lower temperature settings and chronic O3 addition (i.e., A.O3), in water-limited condition. WLCT.O3 = High CO2, higher temperature settings and chronic O3 addition (i.e., CT.O3), in water-limited condition. ns, *, ** and *** mean non-significant, significant at 0.05, 0.01 and 0.001, respectively, Dunnett's test with treatment A as control.

Three European wheat varieties were grown under combinations of abiotic stress factors associated with climate change (see full description and acronyms of treatments in subheading 5.3 in Methods section and in figure footnotes). Analysis of nutrient content of wheat flours from these experiments showed that combinations of abiotic stress conditions did not result in significant changes to C content, a slight reduction in K content and increase in N, Fe, Mg, Mn, P, and Zn. The response varied among the wheat varieties and the treatment combination (Fig. 1). N concentration is a key parameter of grain quality, associated with protein content. N concentration ranged from 1.89 to 1.94 g/100 g dw. In response to the treatments, significantly higher concentrations (between + 3.99 and + 51.92%) were recorded. The varieties responded differently to each treatment: the highest N values in KWS Bittern (+ 43.37% and + 43.63%) were obtained with treatments CT.O3 and T.EpO3, respectively; in Lantvete it was + 50.81% with treatment CT; while in Lennox it was + 51.92% with CT.EpO3. In all the varieties, treatment A.EpO3 lead to the lowest N increase. When each climate parameter was considered alone, highest N values were obtained with chronic O_3_ treatments, followed by episodic and then normal O_3_; N content under high CO_2_ treatments were slightly greater than under ambient CO_2_; treatments with higher temperature showed higher N content than those with lower temperature. Under presence of O_3_ however, high CO_2_ equally affects N content irrespective of the O_3_ regime, while for ambient CO_2_, N content is higher with episodic O_3_, and highest with chronic O_3_. Similarly, higher temperature has a comparable effect on N content irrespective of the O_3_ regime, while the effect of lower temperature is enhanced under episodic O_3_, and more enhanced under chronic O_3_. Water-limited treatment with KWS Bittern slightly reduced N content by − 2.10% to − 4.57%, when compared to their respective controls. But when compared to the control treatment (A), significant increase (p < 0.001) of + 29.31% and + 40.37% were obtained for treatments WLA.O3 and WLCT.O3, respectively. Detailed observations of changes for all the studied minerals are depicted on Fig. 1, Supplementary Figs. 1–8, and data are available in Supplementary Table 1.

### Effect of ozone alone, and in combination with other climatic abiotic stress factors

**Figure 2:**
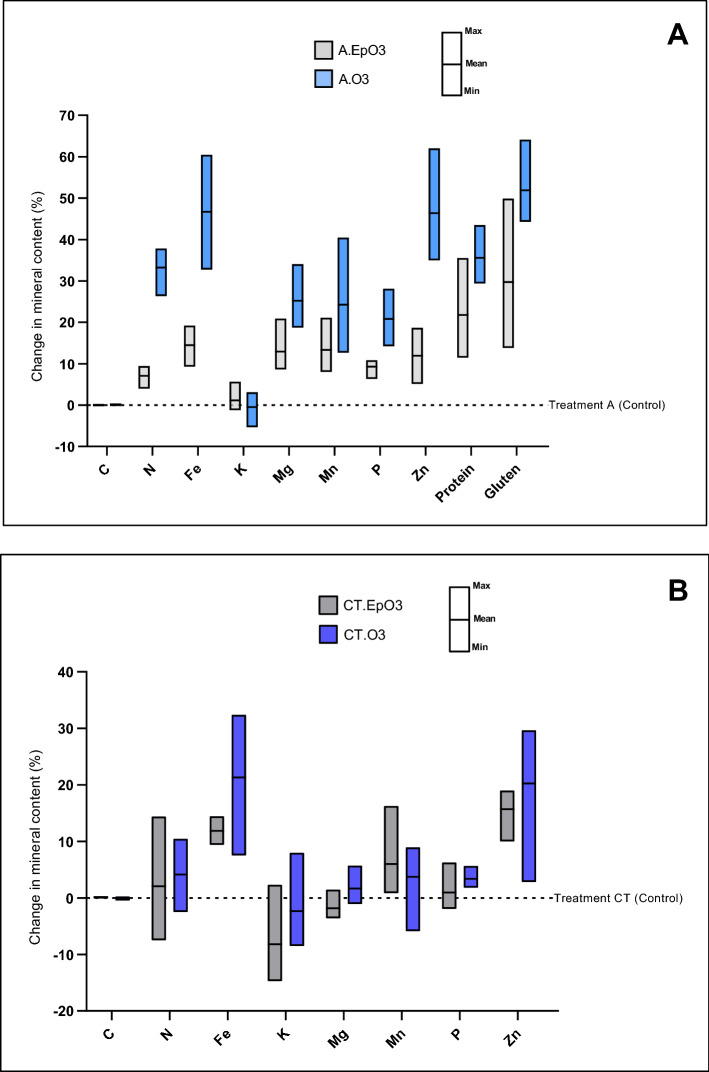
Change of mineral content of three European wheat varieties under effect of ozone alone (**A**) and combined with high CO_2_ and elevated temperature (**B**). A = Ambient CO_2_, lower temperature settings and no O_3_ addition (control). A.EpO3 = Ambient CO_2_, lower temperature settings and episodic O_3_ addition. A.O3 = Ambient CO_2_, lower temperature settings and chronic O_3_ addition. CT = High CO_2_, higher temperature settings, and no O_3_ addition. CT.EpO3 = High CO_2_, higher temperature settings and episodic O_3_ addition. CT.O3 = High CO_2_, higher temperature settings and chronic O_3_ addition.

Ozone is one of the most damaging tropospheric air pollutant affecting plant growth and productivity^31,32^ and tropospheric O_3_ concentrations have more than doubled since pre-industrial times^33^. Wheat is sensitive to elevated O_3_ levels, causing differences in grain yields and nutrient content^17^. In this study, treatments with O_3_ alone did not have any noticeable change on C content, marginally reduced K content, but significantly increased the content of N (mean value for the 3 varieties, treatment A.EpO3 =  + 7.10% and treatment A.O3 =  + 33.22%), Fe (+ 14.52% and + 46.73%), Mg (+ 12.94% and + 25.24%), Mn (+ 13.33% and + 24.24%), P (+ 9.29% and + 20.85%), Zn (+ 11.91% and + 46.39%), protein (+ 21.78% and + 35.59%), and gluten (+ 29.73% and + 51.91%); chronic O_3_ increased the content of the nutrients more than episodic O_3_ (Fig. 2A). Comparison of treatments that combine elevated CO_2_ and higher temperature with different O_3_ regimes in this study (CT vs CT.O3 and CT.EpO3) showed that under condition of high CO_2_ and temperature, O_3_ could significantly increase grain contents of Fe (mean value for the 3 varieties, treatment CT.EpO3 =  + 11.86% and treatment CT.O3 =  + 21.32%), Mn (+ 6.02% for CT.EpO3), P (+ 3.37% for CT.O3) and Zn (+ 15.71% for CT.EpO3 and + 20.24% for CT.O3); the effect on the other minerals was not significant; here as well, chronic O_3_ exposure was more effective than episodic (Fig. 2B). However, the effect of these combined climatic factors is not additive: comparing Fig. 2A and B, it appears that the effect of O_3_ on mineral content of wheat is greater in the presence of ambient CO_2_ and lower temperature settings.

### Trade-off between grain yield and nutrient content

**Figure 3:**
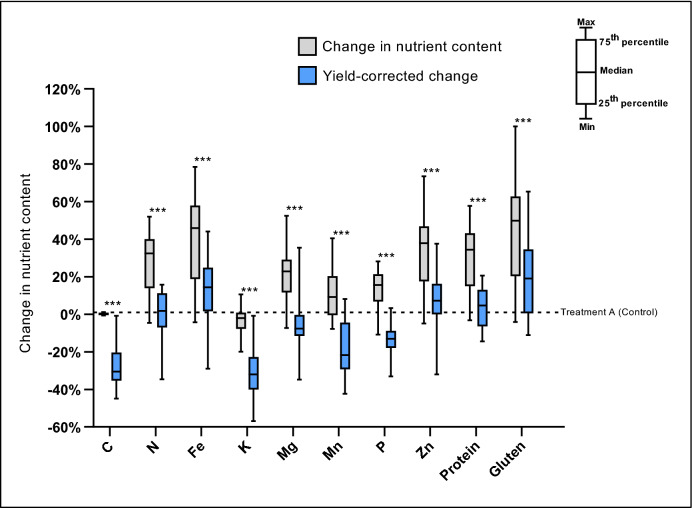
Average percentage change in nutrient content and yield-corrected nutrient content of three European wheat varieties under combined climatic abiotic stress factors, compared to the control. A = Ambient CO_2_, lower temperature settings and no O_3_ addition (control). ***Indicates significant difference at *p* = 0.001 between the normal and the yield-corrected nutrient content, one-tailed pairwise Student’s t-Test.

All treatments caused a decrease in yield^29^ from − 14 to − 36% in KWS Bittern, − 26 to − 46% in Lantvete, and − 16 to − 37% in Lennox^29^, the resultant effect on nutrient yield (mass of grain nutrient per unit area) shows a correction towards significantly lower values to various extents, depending on the initial content. The overall impact of abiotic stress treatments on nutrient yield was positive for gluten, Fe, Zn and protein, with an increase of + 19.11%, + 14.42%, + 7.20% and + 4.60%, respectively, while the decrease of yield counterbalanced the gain in concentration of the other nutrients, resulting in decrease of K (− 32.08%), Mn (− 21.65%), P (− 13.12%), and Mg (− 7.66%) (Fig. 3).

**Figure 4:**
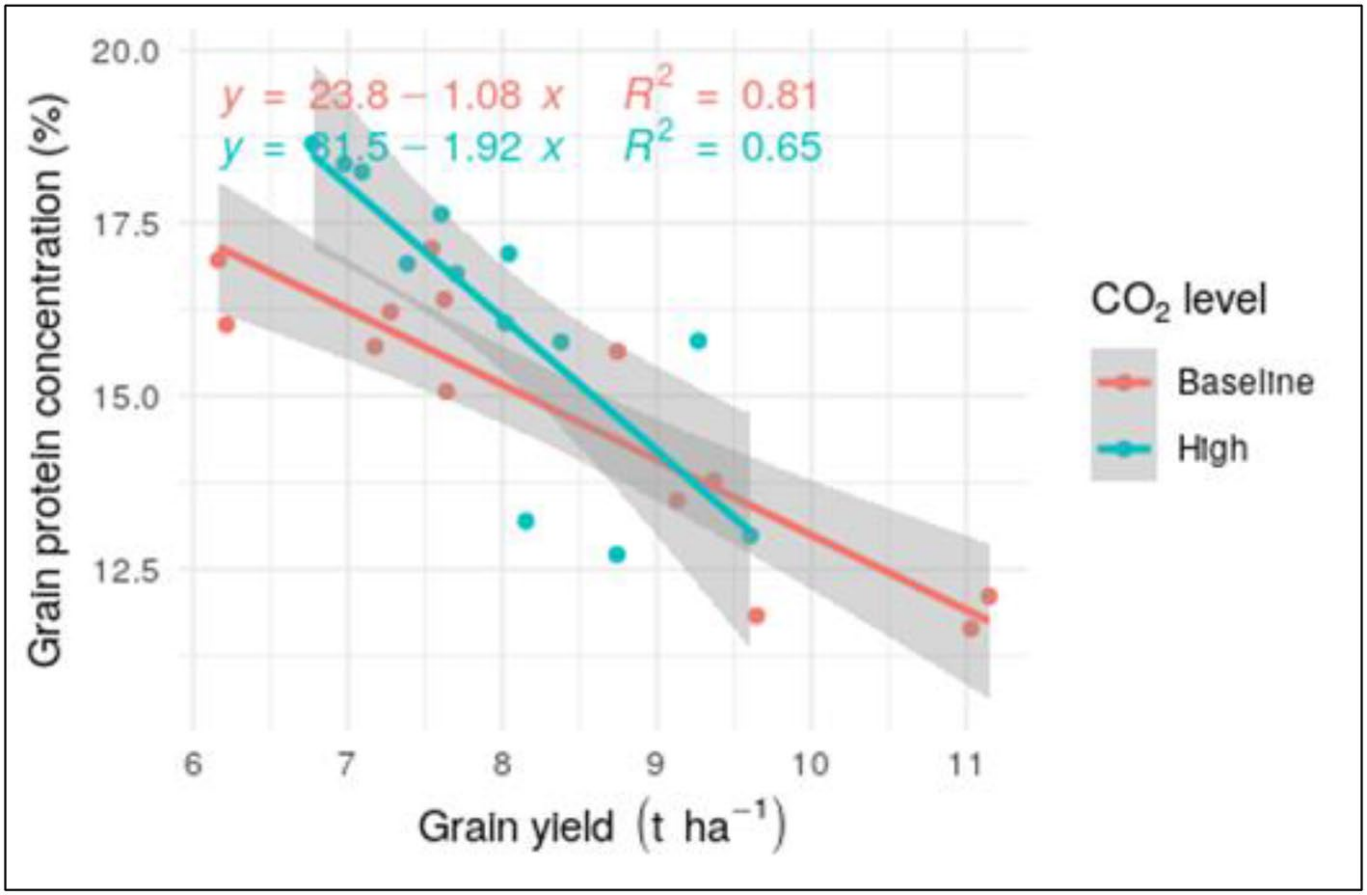
Relationship between grain yield and protein content for spring wheat varieties KWS Bittern, Lennox and Lantvete grown under baseline CO_2_ (400 ppm; red points) and high CO_2_ level (700 ppm; blue points). Solid red and blue lines are linear regressions fitted against the red and blue points respectively and grey areas are 95% confidence intervals.

The decline in protein content was significantly higher under future CO_2_ conditions in comparison with the same plants grown with present-day CO_2_. We modelled the changes in protein under different CO_2_ conditions, to understand how it would be influences by changes in yield. Our results indicate that under present-day CO_2_ level, protein content declines by − 1.08% for 1 t/ha increase in yield, regardless of other stress conditions (low/high temperature, fully irrigated/water stressed, episodic/chronic exposure to O_3_) or the wheat cultivar exposed (Fig. 4). Yield explained a lower percentage of the variance in protein content under high CO_2_. Hence, factors that are not related to atmospheric CO_2_, such as genotypic differences between the cultivars may become increasingly important for the determination of protein content under future climate change conditions.

### Effect of combined abiotic stress factors on the ability of wheat to meet nutrient needs

**Figure 5:**
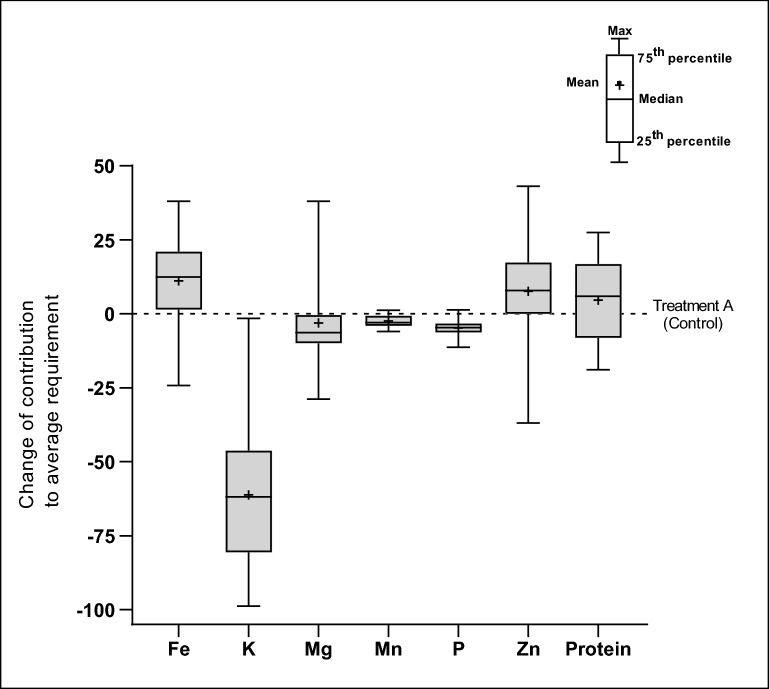
Contribution of average wheat supply of 298.5 g/person/day to daily average requirement of some essential nutrients of European adults (≥ 18 years). Treatment A = Ambient CO2, lower temperature settings and no O3 addition (control).

Using yield-corrected nutrient contents, we estimated the contribution of the global average wheat supply 298.5 g/person/day to the average nutrient requirement of European adults (Fig. 5 and Supplementary Fig. 9). The resulting effect of combined abiotic factors would increase the ability of wheat to meet adult daily dietary requirements by + 6% to + 12% for protein, Zn and Fe, but decrease those of Mg, Mn, and P by − 3% to − 6%, and K by − 62%.

## Discussion

Global diets are highly reliant on cereals, most particularly rice, wheat, and maize. Any change in yield or quality can affect the dietary health of millions of people. Our results from controlled chamber experiments offer robust evidence that combinations of abiotic stresses associated with climate change affect the yield and nutrient content of wheat, supporting earlier observations and modelling. For most nutrients, the European landrace variety Lantvete outperformed the commercial cultivars This agrees with other studies which showed that landraces recorded higher nutrient content than cultivars, for twelve nutritionally important minerals^10^. Lantvete showed grain yield plasticity across climate treatments, indicating a trend of not losing yield under abiotic stress conditions^29^. Varietal diversity has been demonstrated in wheat in response to elevated O_3_^17,37^ and against heat stress^13^. The variation of reduction of yield under elevated temperature across wheat cultivars in South Africa suggested that global warming impacts may be mitigated through the sharing of gene pools among wheat breeding programs^38^. These differences of response between cultivars offer a good opportunity for breeding towards more climate-resilient and nutritious crops^14^.

All abiotic stress conditions reduced yield of wheat, but surprisingly, considering yield trade-offs, combined abiotic stresses could actually increase the contribution of wheat towards meeting Fe, Zn and protein requirements by + 12.44%, + 7.91% and + 5.88%, respectively. Conversely, the combined stresses could decrease the contribution by − 2.87% to − 6.36% for Mg, Mn and P, and − 61.80% for K requirements. Although one should consider that nutrient losses occur during storage and processing of grain. For example, there is an approximate 25% loss of protein, 90% loss of Mn, 85% loss of Zn, and 80% loss of Mg, K and Cu when wheat is milled and refined into flour^61^, and a 1.7% to 4.6% loss of Cu, Mn, Fe and Zn during 60-day storage and baking of wheat flour^62^.

Ozone had particularly interesting effects on micronutrient accumulation. Elevated O_3_ (as a single stress) has been shown to affect nutrient content of grains^17,18,32^, and there is a high variability of sensitivity to tropospheric ozone among species and cultivars^17,34^. Ozone enters in the plant through the stomata of the underside of the leaf, and most likely reacts with molecules in the cell wall, and due to its strong oxidative property, it triggers production of reactive oxygen species (ROS). The ROS damage cellular components, resulting in reduction of photosynthesis and other important physiological functions, acceleration of leaf senescence, and reduction of plant growth, with the consequence of weaker plants and impaired yield attributes^35–37^. On the other hand, O_3_-stressed plants maintain to a larger extent N uptake while biomass accumulation is reduced, resulting in an increased grain protein content^38^. Furthermore, increase of grain mineral concentration may be attributed to a higher accumulation of these minerals, as a non-enzymatic antioxidant defense responses to abiotic stress^39^. This could explain why in our study, plants under chronic O_3_ exposure concentrated more minerals in their grain than the episodic O_3_-exposed plant. Furthermore, the effect of drought stress on wheat minerals was reverted under chronic O_3_ exposure, while changes resulting from high temperature overrode the changes due to CO_2_ and O_3_^29^. For adaptation of crops to abiotic stress, selection of cultivars resistant to multiple abiotic stresses have been recommended^41^. Further work is needed to elucidate the molecular mechanisms underpinning these observations.

Average decreases of − 1.12% and − 1.2% respectively in wheat grain protein content for 1 t/ha increase in grain yield were reported in previous studies^42,43^. Our results are in line with the above studies. With regards to modelling the effect of O_3_ on protein content, a simple statistical relationship between grain yield and protein content may provide an efficient parameterization. Under plant exposure to high O_3_, the observed increase in protein content is a result of the decrease in grain yield^43^, which is already simulated in some crop models^44–46^. It should be noted, however, that Eichi et al.^47^ found that the relationship between grain protein content and yield for wheat in Australia varies between low and high productivity environments. Hence, the linear regression of Fig. 5 may not be extrapolated with any degree of confidence below or above certain yield levels. Overall, our results agree with previous studies that the negative relationship between grain protein content and yield becomes stronger under elevated CO_2_^48,49^, and a single linear regression based on yield may become less efficient in predicting protein content of wheat grain. Nevertheless, combined abiotic stress conditions associated with climate change are not likely to affect protein intakes to a great extent.

Intake of certain micronutrients are not met in many European countries. A study on adult nutrient intakes from national dietary surveys of European populations showed that although all countries met the female and male WHO recommended nutrient intakes (RNIs) for Zn, intakes of Fe, I and K were poorly attained in women^50^. Our results suggest that plant responses to abiotic stresses associated with climate change could contribute to mitigating inadequacy of Fe and potentially Zn from wheat consumption but could exacerbate K deficiency. This highlights the precarity of the global food system which relies so heavily on very few crops for human nutrition. Further research needs to model nutrient intake for high and low wheat consumers, a rising population and take into account nutrient losses during processing. Furthermore, we recommend that micronutrient analyses are included alongside yield and protein levels in field experiments with different varieties and geographical locations.

## Conclusion

The results from our controlled-chamber experiments indicate that combined abiotic stress factors associated with climate change negatively affect yield, but effects on protein and most micronutrients (apart from K) may compensate for yield losses. Chronic exposure to O_3_ had interesting effects which appear to counteract the effects of CO_2_, heat, and drought. This may be due to the role of some micronutrients in ozone-induced physiological responses. Cultivar differences suggest that there are genetic differences that could be explored through breeding of climate-resilient and nutritious crops.

## Methods

### Plant materials

Three spring wheat varieties were studied, including two modern varieties (Lennox and KWS Bittern) and one landrace (Sweedish Lantvete). Lennox (Saaten-Union) used in southern France was supplied by Dr. Marie Launay, French National Institute for Agriculture, Food, and Environment (INRAE), Agroclim HDR, France. KWS Bittern used in Denmark was supplied by Danish Agro (Karise, Denmark). The landrace variety (Swedish Lantvete) was available from the Nordic Genetic Resource Center (NordGen), Swedish University of Agricultural Sciences, Alnarp, Sweden. All experiments were carried out according to institutional, national, and international biosafety standards.

Their life cycle is between 3 to 4 months. Twelve seeds of each variety tested were sown in 11 L pots filled with 4 kg of sphagnum (Pindstrup Substrate No. 4, Pindstrup Mosebrug A/S, Ryomgaard, Denmark) and thinned to 8 plants after germination, corresponding to ˜165 plants/m^2^. As the sphagnum was nutrient enriched with 10 g NPK fertilizer (21-3-10, Kemira Denmark A/S), no additional nutrients were added to the pots. Tap water was used for watering. Each variety was represented in each treatment with five pots.

### Climate chamber

The experiment was performed in climate chambers that provided a controlled environment and uniform conditions, thus eliminating other potentially interacting parameters. The facility used was the RERAF phytotron (Riso Environmental Risk Assessment Facility, Technical University of Denmark, Riso, Denmark), which consists of six gastight chambers sized 6 × 4 × 3 m (length, width & height), providing detailed control of temperature, CO_2_, air humidity, light, and O_3_ concentration and exposure duration. Details of description of the climate chamber are available in^24,51,52^.

### Climate abiotic stress treatments

Full details of the experimental conditions and treatments are available in Hansen et al.^29^. Climate change treatments were selected among possible combinations of two present and future temperature levels (19/12 °C or 24/17 °C, both levels simulating days (16 h) that are warmer than nights (8 h)), two concentrations of CO_2_ (400 and 700 ppm), and one of three O_3_ regimes (no O_3_ enrichment, episodic O_3_ exposure, and full-time O_3_ exposure). Ozone concentrations for the treatments without O_3_ enrichment was the climate chambers background levels (5.9 ± 0.5 to 7.2 ± 1.7 ppb), which are lower than the outside average O_3_ concentration near the RERAF phytotron (average of 40.4 ppb, and maximum 1 h concentrations between 70.9 and 86.6 ppb). For both the episodic and full-time O_3_ exposure treatments, O_3_ concentration target was 80–100 ppb during the day (16 h of daytime O_3_ exposure), and chamber background level equivalent to the no O_3_ enrichment treatments at night. The full-time O_3_ exposure treatments started at sowing, while the episodic O_3_ exposure treatments began when Lennox variety reached Zadoks’ developmental stage 31 (ZS31—first node detectable) and ended when the variety reached stage 69 (ZS69—anthesis complete)^53^.

Throughout the experiment, relative humidity was maintained at 55/70% (day/night) for all treatments. To provide appropriate supply of water, plants were watered 3 times a week. All plants received increasingly more water as they grew, the warm treatment plants were, by design and by consumption, given more water than ambient treatment plants. Pots were weighed before and after watering to ensure the same amount of water was accessible in the treatment regardless of the pot’s previous consumption. Additionally, a water-limited (WL) treatment was given to 2 selected climate combination treatments of variety KWS Bittern. It consisted of limited water supply in A.O3 and CT.O3, where the plants were subjected to chronic O_3_ addition, in different CO_2_ and temperature conditions. Thus, 10 climate treatment combinations were tested in total and named as follows:

- A = Ambient CO_2_, lower temperature settings and no O_3_ addition (control)
- A.EpO3 = Ambient CO_2_, lower temperature settings and episodic O_3_ addition
- A.O3 = Ambient CO_2_, lower temperature settings and chronic O_3_ addition
- C.EpO3 = High CO_2_, lower temperature settings and episodic O_3_ addition
- CT = High CO_2_, higher temperature settings, and no O_3_ addition
- CT.EpO3 = High CO_2_, higher temperature settings and episodic O_3_ addition
- CT.O3 = High CO_2_, higher temperature settings and chronic O_3_ addition
- T.EpO3 = Ambient CO_2_, higher temperature and episodic O_3_ addition
- WLA.O3: Ambient CO_2_, lower temperature settings and chronic O_3_ addition (i.e., A.O3), in water-limited condition
- WLCT.O3 = High CO_2_, higher temperature settings and chronic O_3_ addition (i.e., CT.O3), in water-limited condition.

Process values of treatment parameters such as relative humidity, CO_2_ concentration, and temperature were logged by a data collection system several times per minute. The O_3_ concentration was monitored twice every hour. At maturity, with moisture content around 9–13%, grains were harvested, threshed and winnowed, and the grains from plants of each replicate of treatment was mixed for further analyses.

### Nutrient analysis

Grains were pulverized into whole wheat flour using a household blender, 600 mg of flour was weighed in a glass test tube, 3 mL of 69% HNO_3_ (Hiperpur, Panreac, Spain) and 2 mL of deionized water (Milli-Q, Merck, Spain) were added. The mixture was digested in a microwave (Milestone, Ultrawave, Italy) at 240 °C and 40 bar for 40 min at 1500 W, and the digesta was brought to a final volume of 50 mL with Milli-Q water. Minerals (C, N, F, K, Mg, Mn, P, and Zn) were analyzed using inductively coupled plasma optical emission spectroscopy (ICP-OES). Analysis was performed on a PerkinElmer, Optima 4600 DV ICP-OES analyzer (Waltham, USA). The running parameters were set as follow: plasma flow 15 L/min, auxiliary flow 0.2 L/min, nebulizer flow 0.8 L/min, power 1300 W, reading distance 15 mm, reading position radial (K) and axial (Mg, Mn, Zn, Fe and P), integration time 5–10 s, and number of replicas 3. For quantification, standards (Panreac Química SLU, Spain) were prepared in HNO3-H2O in the same proportion as the samples (matrix matched calibration standards). Wheat standard reference material GBW10011 was used for recovery and limits determination. The detection and quantification validation parameters are summarized in Table 1. Nutrient content was corrected from grain moisture content, determined by using the Association of Analytical Communities (AOAC) Method 991.39^54^. Content of N and C were expressed in g/100 g dry weight (dw), while Fe, K, Mg, Mn, P and Zn were in mg/100 g dw. Gluten content was determined according to the ICC 155 procedure^55^ by the Nordic Seed Laboratory Services, and expressed in percent of protein content. Protein content was obtained by multiplying the nitrogen content by 5.83^56^ and expressed in g/100 g dw.

**Table 1:** Detection and quantification parameters of wheat minerals by ICP-OES.

Mineral	K	Mg	Mn	Zn	Fe	P
ICP-OES wavelengths (nm)	766.49	285.213	257.61	206.2	238.204	213.617
Standard concentration range	0.5–50	0.5–50	1–100	10–1000	10–1000	1–50
Standard concentration unit	mg/L	mg/L	µg/L	µg/L	µg/L	mg/L
Linearity	0.9999	0.9999	1.0000	0.9998	1.0000	0.9995
Recovery (%)	93.7	99.8	74.0	96.8	65.9	68.7
RSD (%)	6.4	6.5	5.9	7.6	6.1	6.2
LOD (mg/kg)	0.8111	0.0262	0.00276	0.0514	0.00178	2.604
LOQ (mg/kg)	36.198	0.173	0.00779	0.3098	0.2448	17.11

### Impact on future food and nutrition security

To evaluate the overall effect of the treatments on grain nutrients availability, yield data from the experiment were obtained from Hansen et al.^29^. Yield data of each treatment was used to correct the value of content of each nutrient, and the yield-corrected nutrient content were compared with the original ones. The potential repercussions of effect of both climate treatments and yield on food and nutrition security under future climate scenarios was analyzed with a case study of European adults. For this, per capita wheat supply (298.55 g/day) was obtained from FAO food supply data for Europe^8^. Daily average requirements (AR) of each nutrient were obtained from EFSA Dietary Reference Values for the EU database^57^ for adults (≥ 18 years) males, and females not under any physiological status (pregnant, lactating, menopausal). For Zn, values at high level of phytate intake (LPI = 1200 mg/day) were considered. These were combined with the yield-corrected nutrient content to estimate the potential percent contribution of each nutrient to average daily intake of some essential nutrients of adults in Europe.

### Data analysis

Climate chamber experiments were performed in triplicate, and each wheat sample was analyzed in triplicate. Data were statistically analyzed using IBM SPSS Statistics v 26. ANOVAs were performed to determine the effect individual and combination of treatment on the content of each nutrient, one-way Dunnett's test with treatment A as control was applied for temperature, CO_2_ and O_3_ treatments. For drought experiment, each water-limited (WL) treatment was also compared against its corresponding control, i.e., WLCT.O3 vs CT.O3 and WLA.O3 vs A.O3. Additionally, performance of landrace variety Lantvete was compared with that of modern varieties KWS Bittern and Lennox using a 1-tailed pairwise Student's t-test. Similarly, a 1-tailed pairwise Student's t-test was also used to assess the significance of differences between original nutrient contents and yield-corrected nutrient contents. Three levels of significance (0.05, 0.01 and 0.001) were considered. The trade-off between grain yield and protein content was quantified using linear regressions of the three cultivars growing under baseline CO_2_ (400 ppm) and future CO_2_ (700 ppm) levels; the grain yield data were converted to t/ha^46^. Graphical representations were generated using R version R-4.0.1 and GraphPad Prism version 9.0.2 for Windows.

## Supplementary Information

Supplementary Figures.

Supplementary Tables.
