# Supplementary material for: Effects of combined abiotic stresses on nutrient content of European wheat and implications for nutritional security under climate change
Source: Sci Rep. 2022 Apr 5;12:5700. doi: 10.1038/s41598-022-09538-6 (PMC8983673; doi:10.1038/s41598-022-09538-6)
Supplement: Supplementary file 1 — Supplementary Figures. [file 41598_2022_9538_MOESM1_ESM.docx]

**Supplementary Figures 1-8:** Interaction effects of temperature, CO2, O3 and water availability on mineral and nutrient content of three spring wheat varieties.


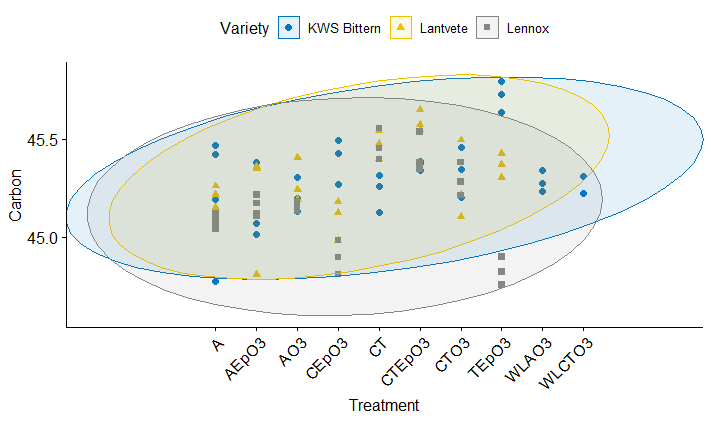

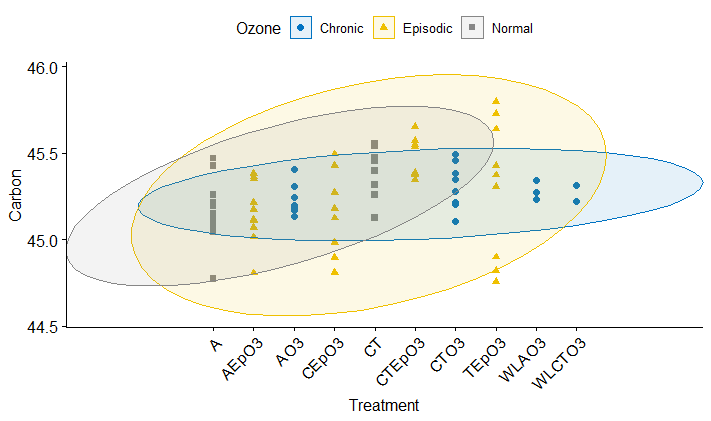


**B**

**A**


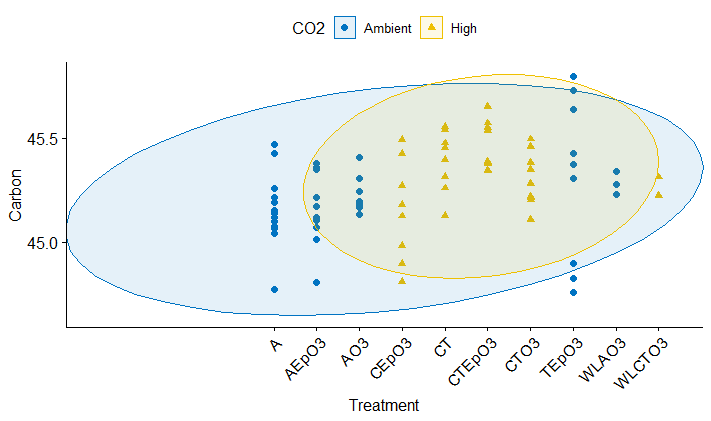

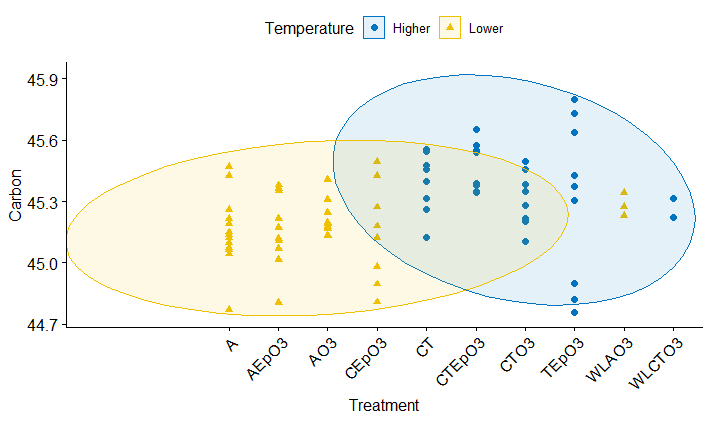

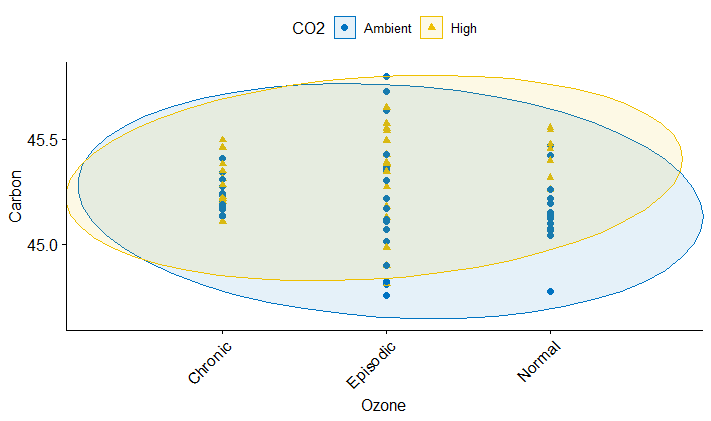

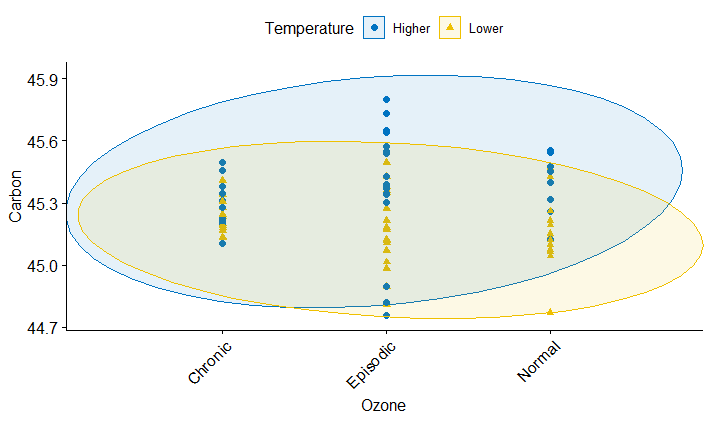


**F**

**D**

**C**

**E**

**D**

**C**

**Supplementary Figure 1.** Effect of temperature, CO2, ozone and water availability on carbon content of wheat.

A = Ambient CO2, lower temperature settings and no O3 addition (control). A.EpO3 = Ambient CO2, lower temperature settings and episodic O3 addition. A.O3 = Ambient CO2, lower temperature settings and chronic O3 addition. C.EpO3 = High CO2, lower temperature settings and episodic O3 addition. CT = High CO2, higher temperature settings, and no O3 addition. CT.EpO3 = High CO2, higher temperature settings and episodic O3 addition. CT.O3 = High CO2, higher temperature settings and chronic O3 addition. T.EpO3 = Ambient CO2, higher temperature and episodic O3 addition. WLA.O3: Ambient CO2, lower temperature settings and chronic O3 addition (i.e., A.O3), in water-limited condition. WLCT.O3 = High CO2, higher temperature settings and chronic O3 addition (i.e., CT.O3), in water-limited condition.


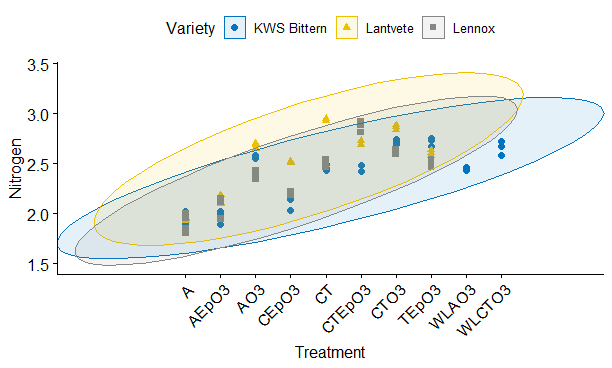

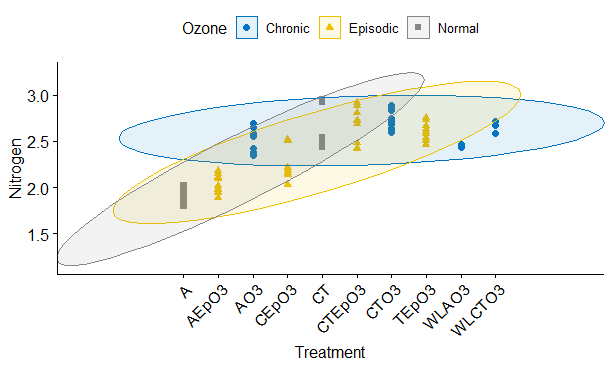


**B**

**A**


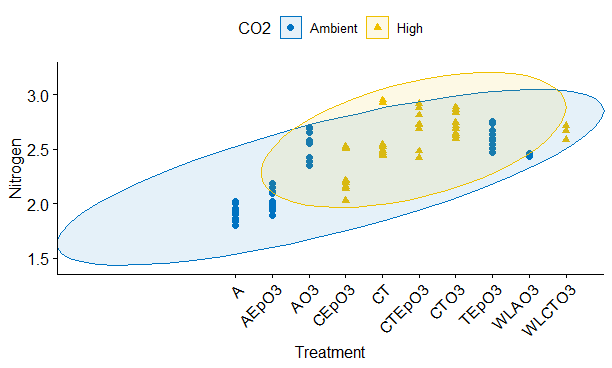

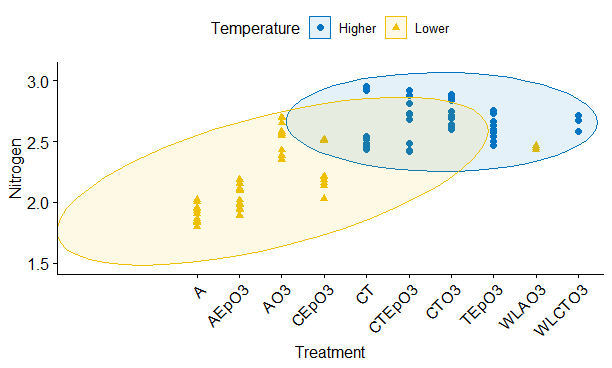


**D**

**C**


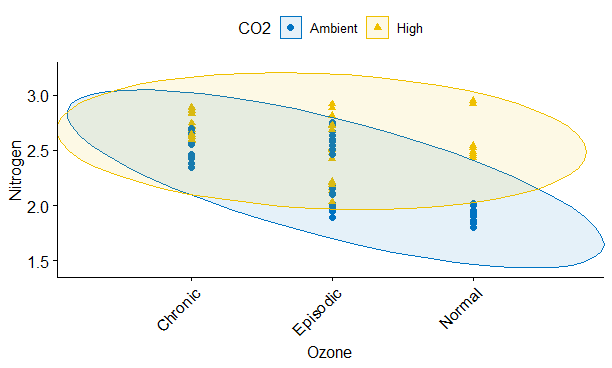

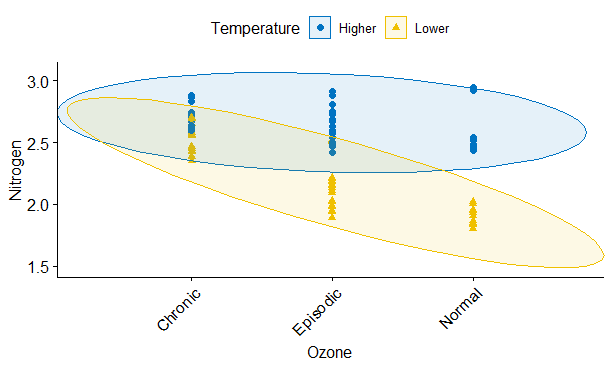


**F**

**E**

**Supplementary Figure 2.** Effect of temperature, CO2, O3 and water availability on nitrogen content of wheat.

A = Ambient CO2, lower temperature settings and no O3 addition (control). A.EpO3 = Ambient CO2, lower temperature settings and episodic O3 addition. A.O3 = Ambient CO2, lower temperature settings and chronic O3 addition. C.EpO3 = High CO2, lower temperature settings and episodic O3 addition. CT = High CO2, higher temperature settings, and no O3 addition. CT.EpO3 = High CO2, higher temperature settings and episodic O3 addition. CT.O3 = High CO2, higher temperature settings and chronic O3 addition. T.EpO3 = Ambient CO2, higher temperature and episodic O3 addition. WLA.O3: Ambient CO2, lower temperature settings and chronic O3 addition (i.e., A.O3), in water-limited condition. WLCT.O3 = High CO2, higher temperature settings and chronic O3 addition (i.e., CT.O3), in water-limited condition.


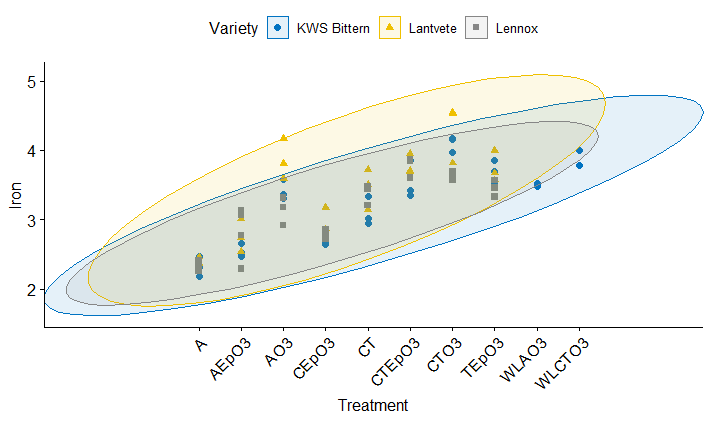

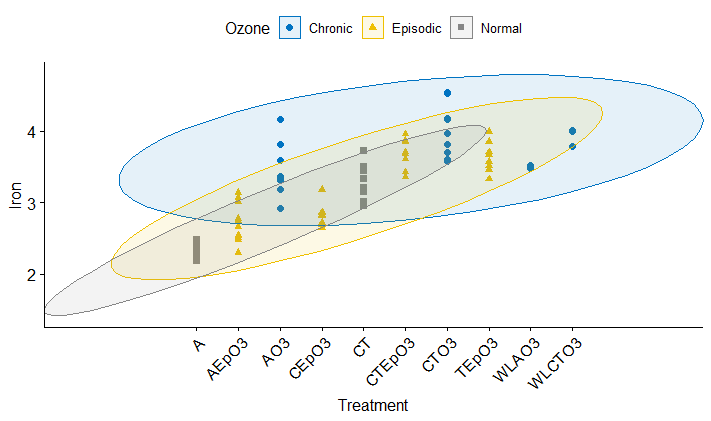


**B**

**A**


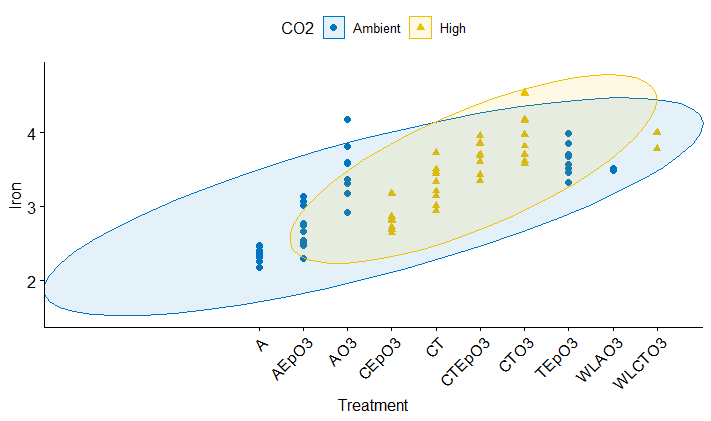

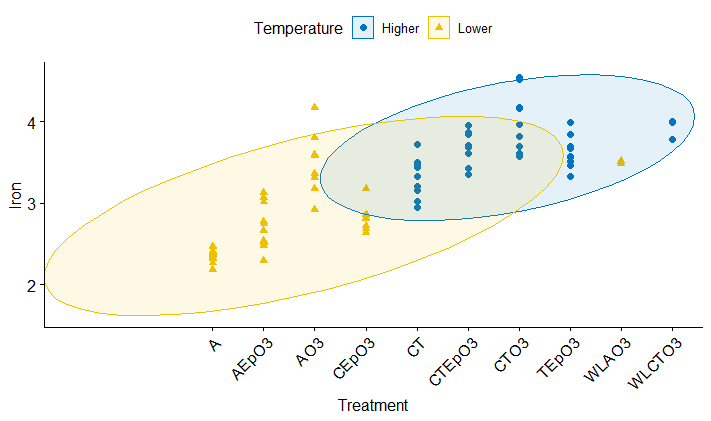


**D**

**C**


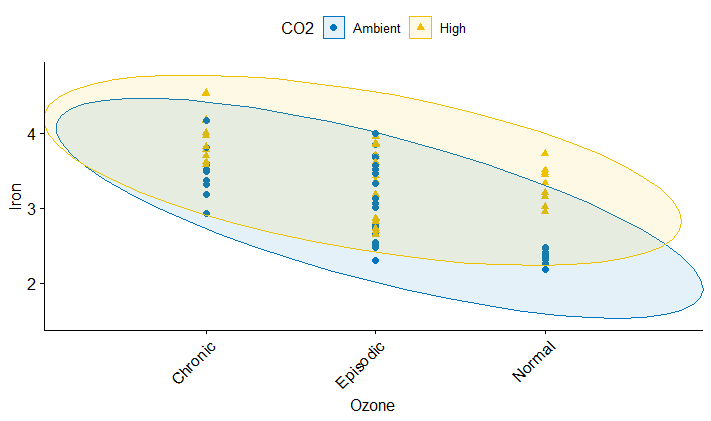

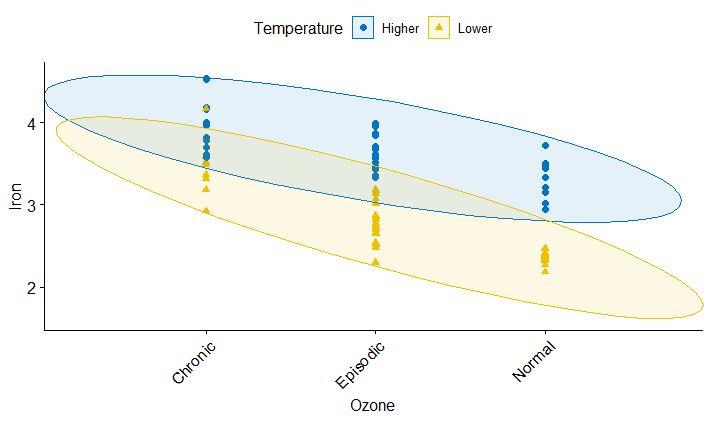


**F**

**E**

**Supplementary Figure 3.** Effect of temperature, CO2, O3 and water availability on iron content of wheat.

A = Ambient CO2, lower temperature settings and no O3 addition (control). A.EpO3 = Ambient CO2, lower temperature settings and episodic O3 addition. A.O3 = Ambient CO2, lower temperature settings and chronic O3 addition. C.EpO3 = High CO2, lower temperature settings and episodic O3 addition. CT = High CO2, higher temperature settings, and no O3 addition. CT.EpO3 = High CO2, higher temperature settings and episodic O3 addition. CT.O3 = High CO2, higher temperature settings and chronic O3 addition. T.EpO3 = Ambient CO2, higher temperature and episodic O3 addition. WLA.O3: Ambient CO2, lower temperature settings and chronic O3 addition (i.e., A.O3), in water-limited condition. WLCT.O3 = High CO2, higher temperature settings and chronic O3 addition (i.e., CT.O3), in water-limited condition.


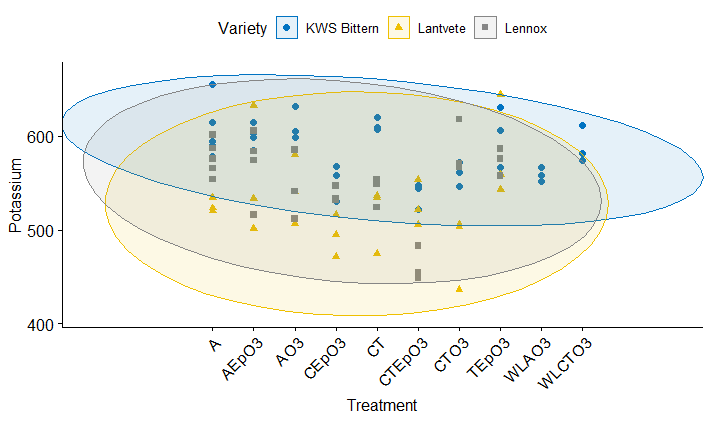

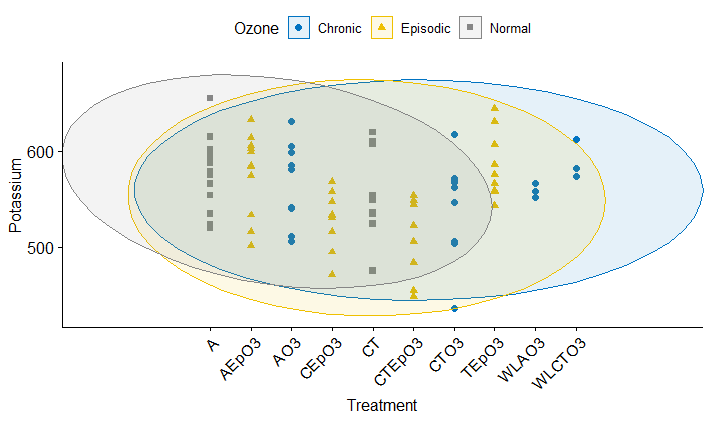


**A**

**B**


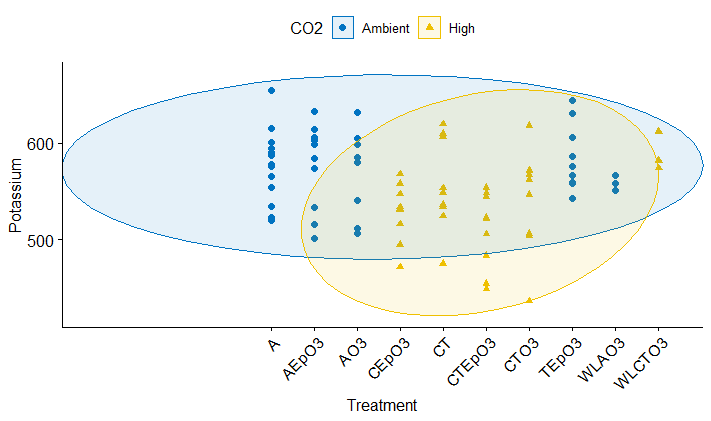

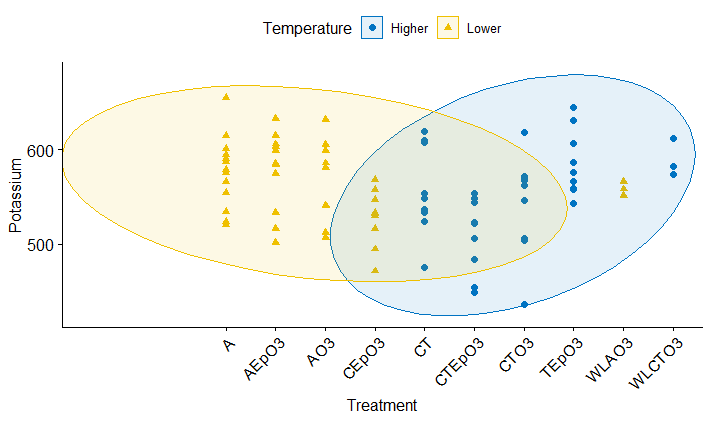


**D**

**C**


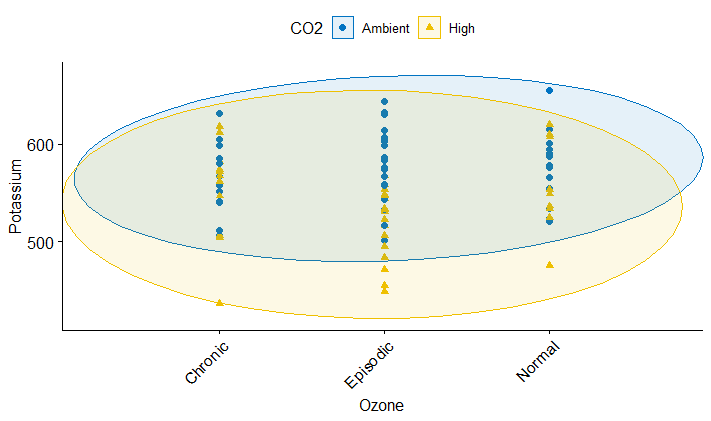

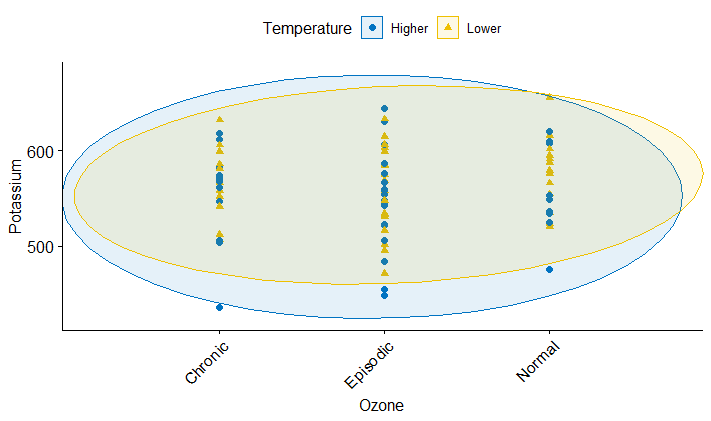


**F**

**E**

**Supplementary Figure 4.** Effect of temperature, CO2, O3 and water availability on potassium content of wheat.

A = Ambient CO2, lower temperature settings and no O3 addition (control). A.EpO3 = Ambient CO2, lower temperature settings and episodic O3 addition. A.O3 = Ambient CO2, lower temperature settings and chronic O3 addition. C.EpO3 = High CO2, lower temperature settings and episodic O3 addition. CT = High CO2, higher temperature settings, and no O3 addition. CT.EpO3 = High CO2, higher temperature settings and episodic O3 addition. CT.O3 = High CO2, higher temperature settings and chronic O3 addition. T.EpO3 = Ambient CO2, higher temperature and episodic O3 addition. WLA.O3: Ambient CO2, lower temperature settings and chronic O3 addition (i.e., A.O3), in water-limited condition. WLCT.O3 = High CO2, higher temperature settings and chronic O3 addition (i.e., CT.O3), in water-limited condition.


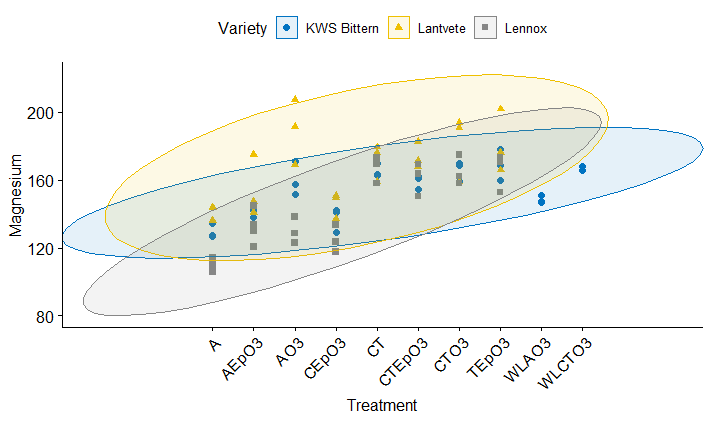

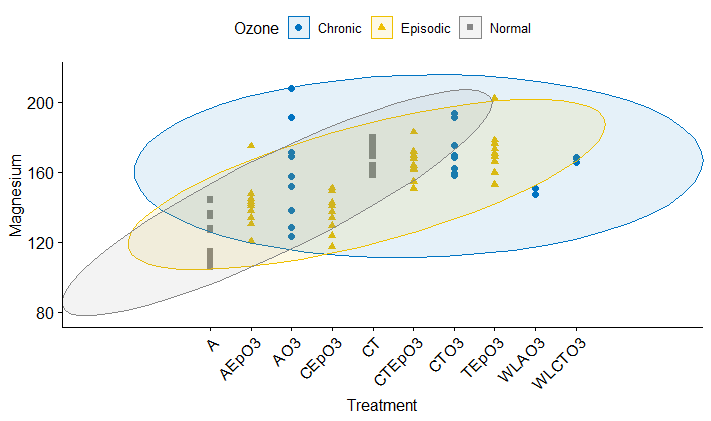


**B**

**A**


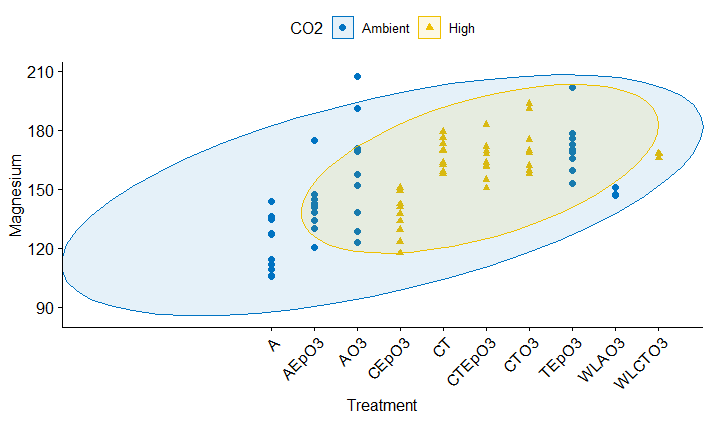

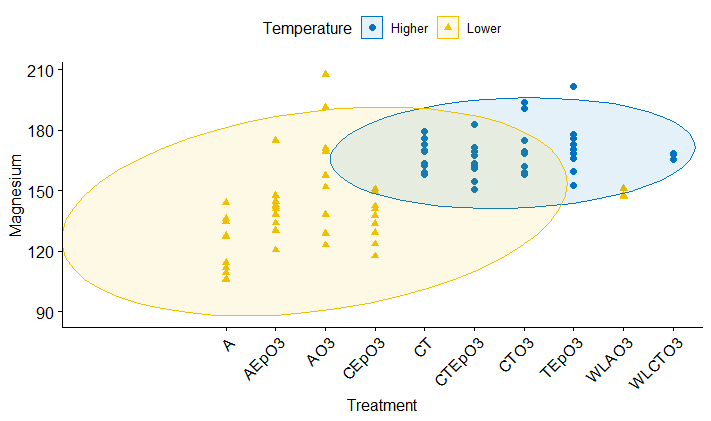


**C**

**D**


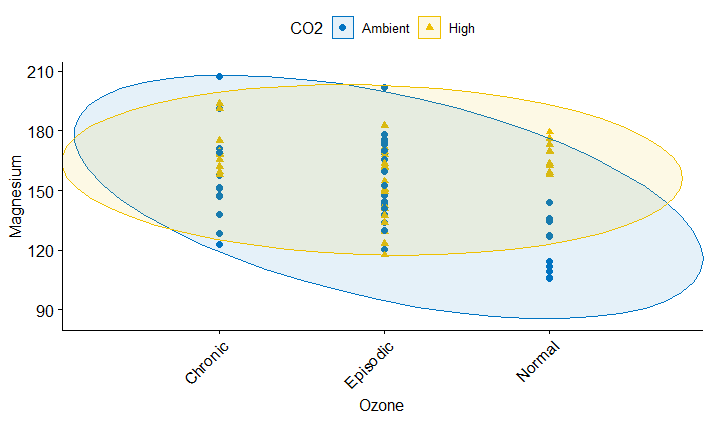

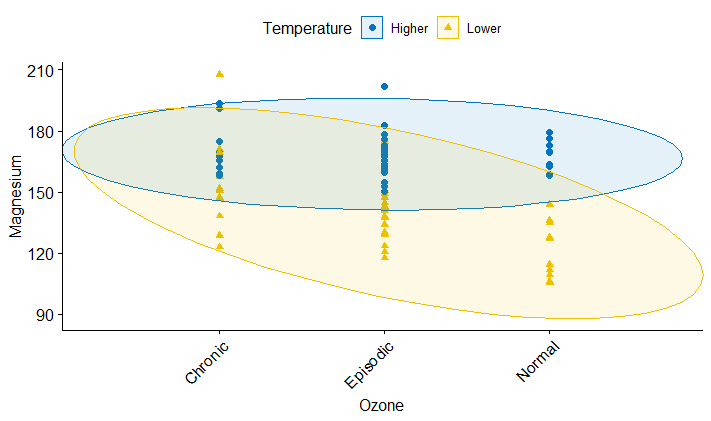


**F**

**E**

**Supplementary Figure 5.** Effect of temperature, CO2, O3 and water availability on magnesium content of wheat.

A = Ambient CO2, lower temperature settings and no O3 addition (control). A.EpO3 = Ambient CO2, lower temperature settings and episodic O3 addition. A.O3 = Ambient CO2, lower temperature settings and chronic O3 addition. C.EpO3 = High CO2, lower temperature settings and episodic O3 addition. CT = High CO2, higher temperature settings, and no O3 addition. CT.EpO3 = High CO2, higher temperature settings and episodic O3 addition. CT.O3 = High CO2, higher temperature settings and chronic O3 addition. T.EpO3 = Ambient CO2, higher temperature and episodic O3 addition. WLA.O3: Ambient CO2, lower temperature settings and chronic O3 addition (i.e., A.O3), in water-limited condition. WLCT.O3 = High CO2, higher temperature settings and chronic O3 addition (i.e., CT.O3), in water-limited condition.


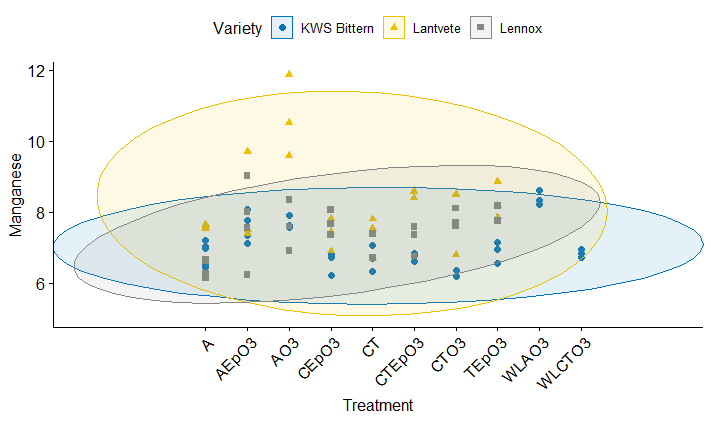

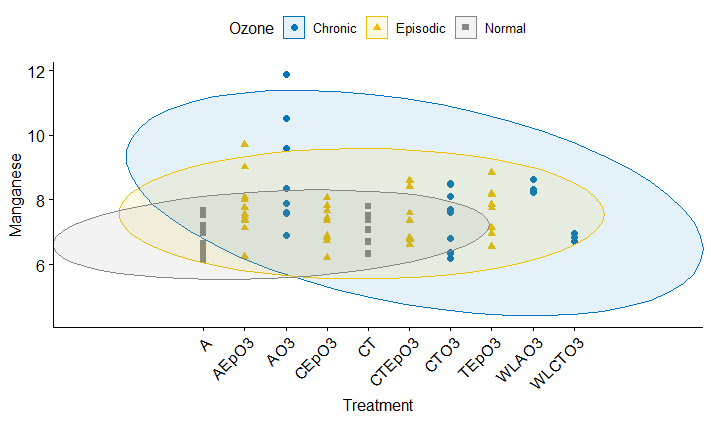


**B**

**A**


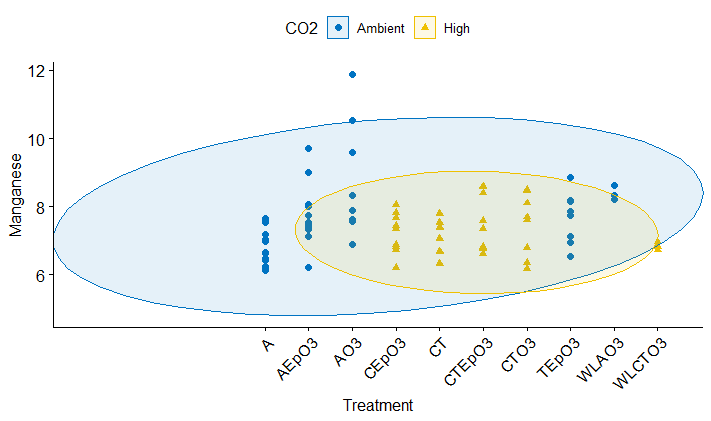

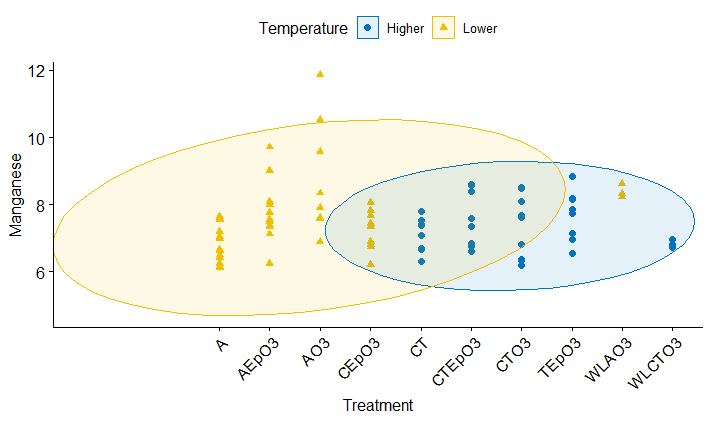


**D**

**C**


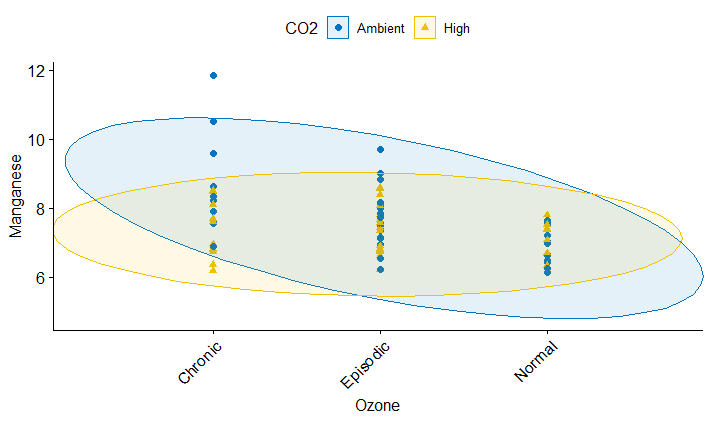

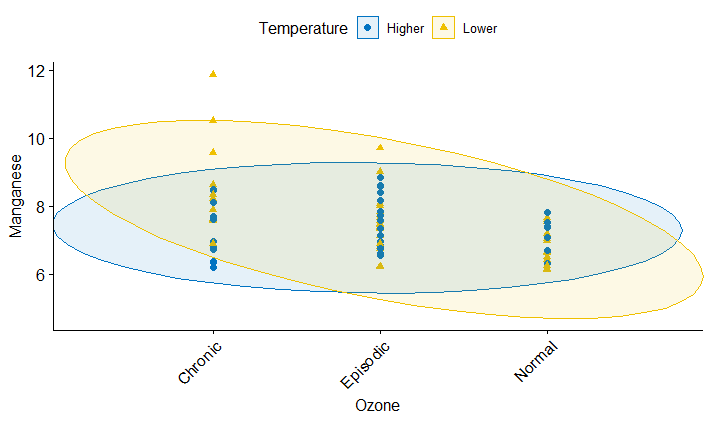


**F**

**E**

**Supplementary Figure 6.** Effect of temperature, CO2, O3 and water availability on manganese content of wheat.

A = Ambient CO2, lower temperature settings and no O3 addition (control). A.EpO3 = Ambient CO2, lower temperature settings and episodic O3 addition. A.O3 = Ambient CO2, lower temperature settings and chronic O3 addition. C.EpO3 = High CO2, lower temperature settings and episodic O3 addition. CT = High CO2, higher temperature settings, and no O3 addition. CT.EpO3 = High CO2, higher temperature settings and episodic O3 addition. CT.O3 = High CO2, higher temperature settings and chronic O3 addition. T.EpO3 = Ambient CO2, higher temperature and episodic O3 addition. WLA.O3: Ambient CO2, lower temperature settings and chronic O3 addition (i.e., A.O3), in water-limited condition. WLCT.O3 = High CO2, higher temperature settings and chronic O3 addition (i.e., CT.O3), in water-limited condition.


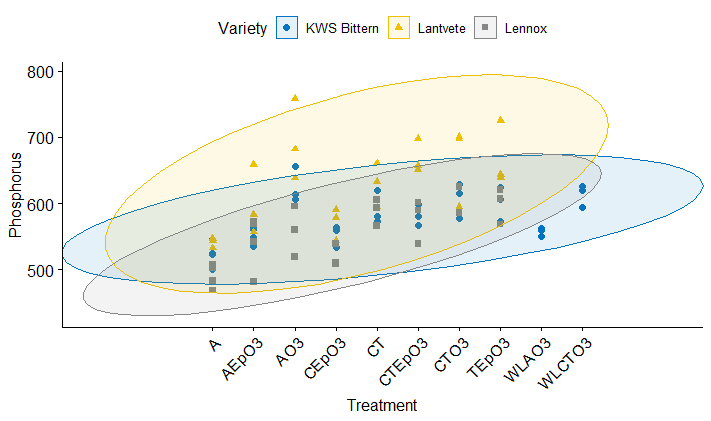

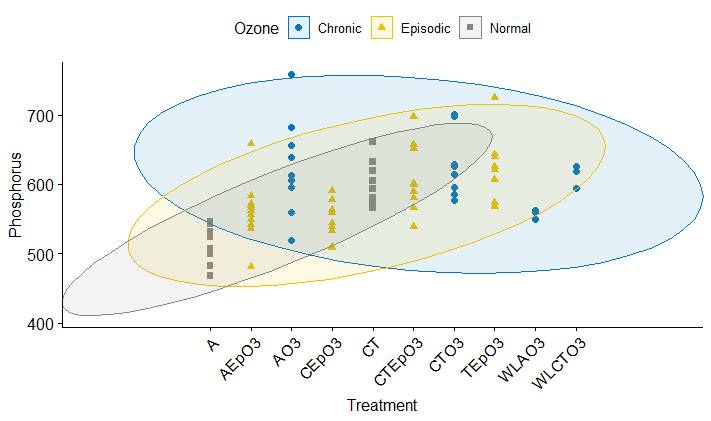


**F**

**E**

**D**

**C**

**B**

**A**


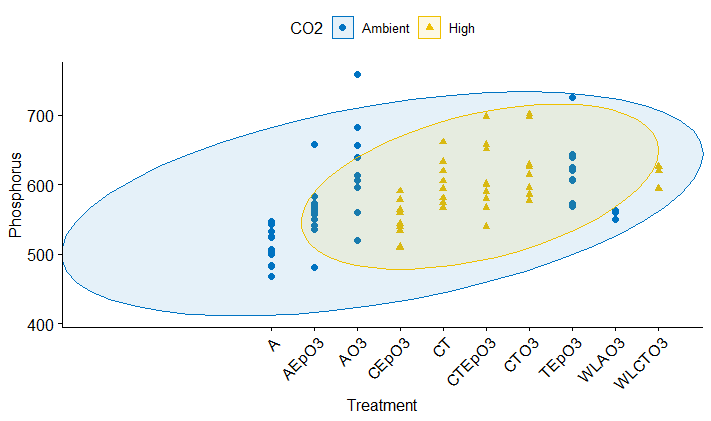

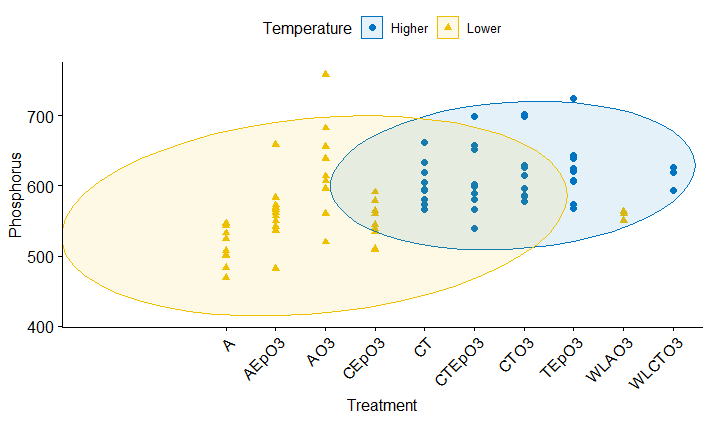


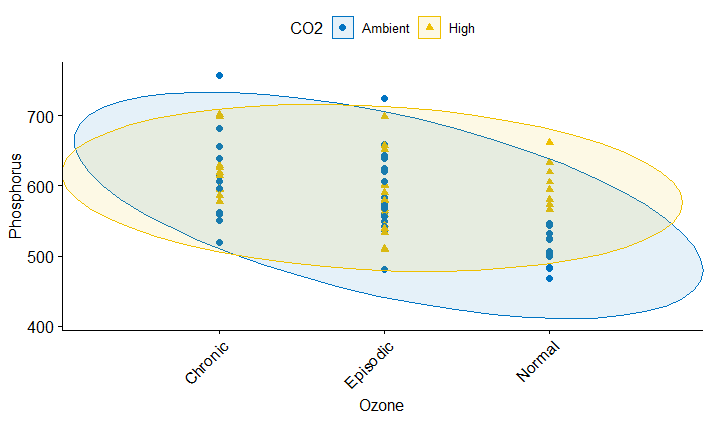

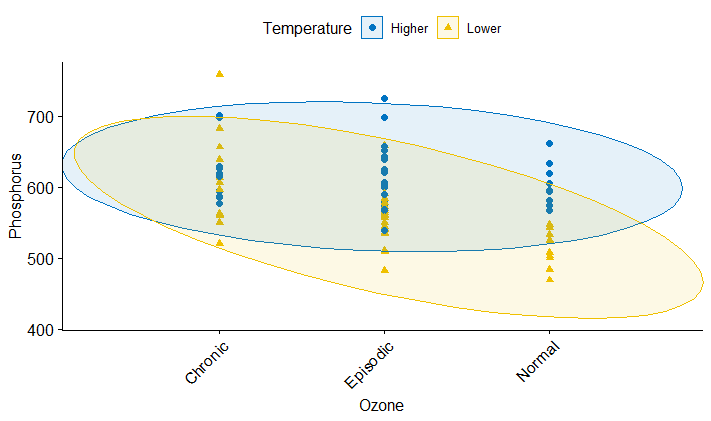


**Supplementary Figure 7.** Effect of temperature, CO2, O3 and water availability on phosphorus content of wheat.

A = Ambient CO2, lower temperature settings and no O3 addition (control). A.EpO3 = Ambient CO2, lower temperature settings and episodic O3 addition. A.O3 = Ambient CO2, lower temperature settings and chronic O3 addition. C.EpO3 = High CO2, lower temperature settings and episodic O3 addition. CT = High CO2, higher temperature settings, and no O3 addition. CT.EpO3 = High CO2, higher temperature settings and episodic O3 addition. CT.O3 = High CO2, higher temperature settings and chronic O3 addition. T.EpO3 = Ambient CO2, higher temperature and episodic O3 addition. WLA.O3: Ambient CO2, lower temperature settings and chronic O3 addition (i.e., A.O3), in water-limited condition. WLCT.O3 = High CO2, higher temperature settings and chronic O3 addition (i.e., CT.O3), in water-limited condition.


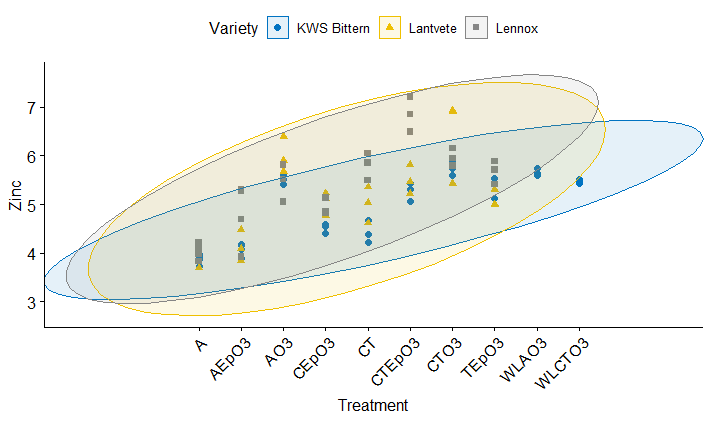

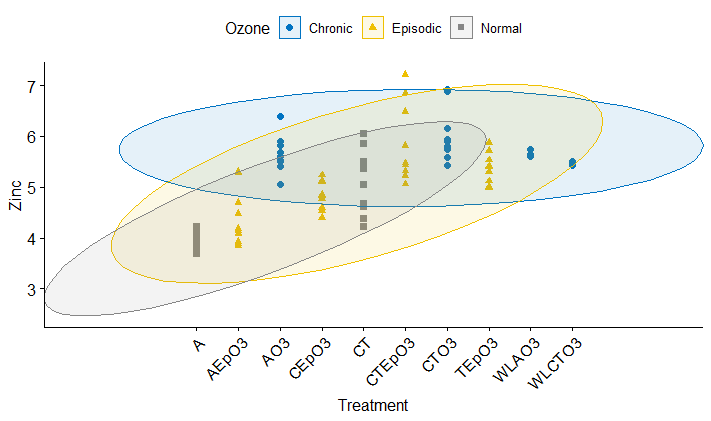


**F**

**E**

**D**

**C**

**B**

**A**


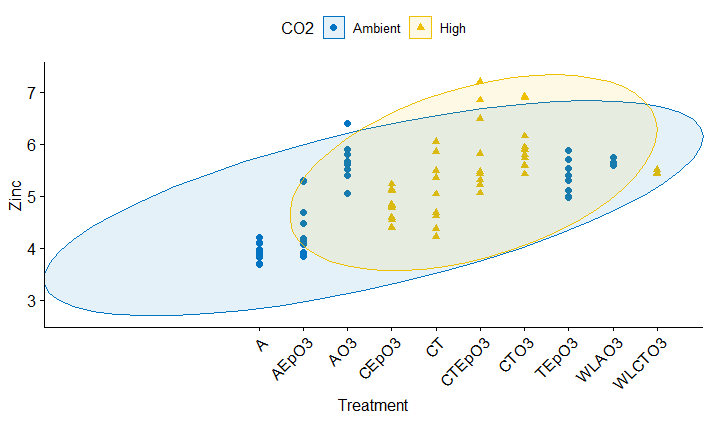

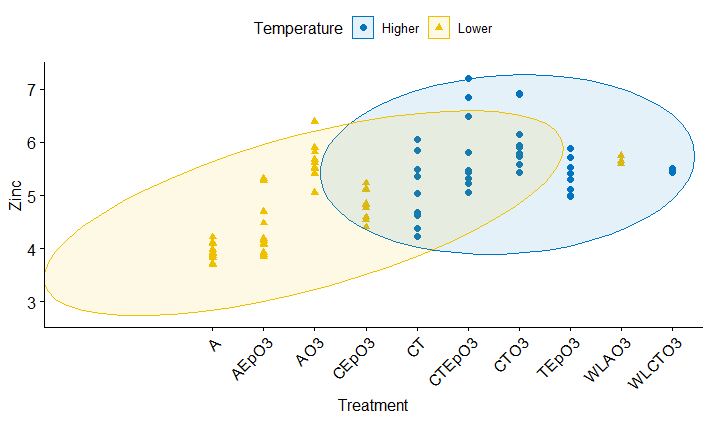


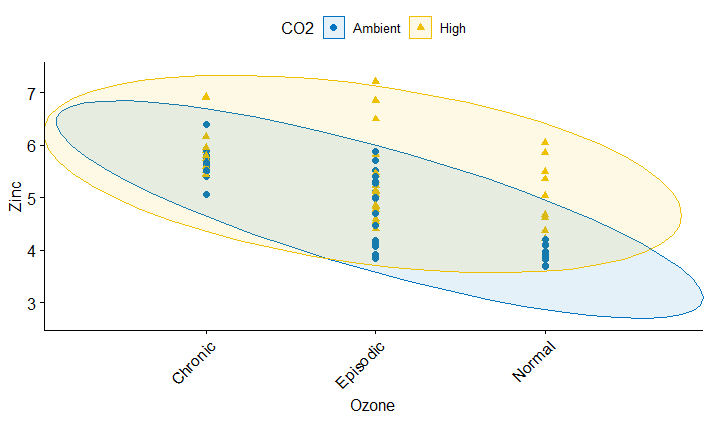

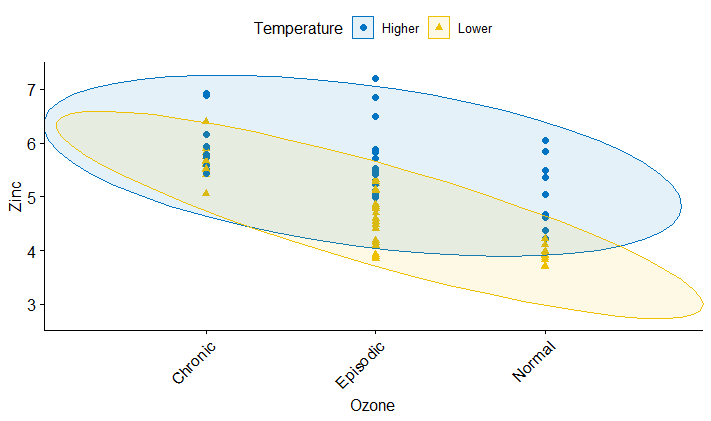


**Supplementary Figure 8.** Effect of temperature, CO2, O3 and water availability on zinc content of wheat.

A = Ambient CO2, lower temperature settings and no O3 addition (control). A.EpO3 = Ambient CO2, lower temperature settings and episodic O3 addition. A.O3 = Ambient CO2, lower temperature settings and chronic O3 addition. C.EpO3 = High CO2, lower temperature settings and episodic O3 addition. CT = High CO2, higher temperature settings, and no O3 addition. CT.EpO3 = High CO2, higher temperature settings and episodic O3 addition. CT.O3 = High CO2, higher temperature settings and chronic O3 addition. T.EpO3 = Ambient CO2, higher temperature and episodic O3 addition. WLA.O3: Ambient CO2, lower temperature settings and chronic O3 addition (i.e., A.O3), in water-limited condition. WLCT.O3 = High CO2, higher temperature settings and chronic O3 addition (i.e., CT.O3), in water-limited condition.

**C**

**B**

**A**

**Supplementary Figure 9.** Effect of temperature, CO2, O3 and water availability on mineral content and contribution to daily average requirement of some essential nutrients of European adults for 3 spring wheat varieties.

A = Ambient CO2, lower temperature settings and no O3 addition (control). A.EpO3 = Ambient CO2, lower temperature settings and episodic O3 addition. A.O3 = Ambient CO2, lower temperature settings and chronic O3 addition. C.EpO3 = High CO2, lower temperature settings and episodic O3 addition. CT = High CO2, higher temperature settings, and no O3 addition. CT.EpO3 = High CO2, higher temperature settings and episodic O3 addition. CT.O3 = High CO2, higher temperature settings and chronic O3 addition. T.EpO3 = Ambient CO2, higher temperature and episodic O3 addition. WLA.O3: Ambient CO2, lower temperature settings and chronic O3 addition (i.e., A.O3), in water-limited condition. WLCT.O3 = High CO2, higher temperature settings and chronic O3 addition (i.e., CT.O3), in water-limited condition.

**Supplementary Table 1.** Results of effect of temperature, CO2, O3 and water availability on grain nutrient content of wheat. (See Excel sheet)
